# Sorghum and Sorghum‐Based Products: Nutritional Composition, Prebiotic Potential and Health Benefits in Gut Microbiota Interactions

**DOI:** 10.1155/ijfo/7084868

**Published:** 2025-11-28

**Authors:** W. M. A. D. Binosha Fernando, Rasheed A. Abdulraheem, Kalmee Pramoda Kariyawasam, Amal Sudaraka Samarasinghe, W. M. Kendrea. T. Fernando, Hannah Barnes, Malina Robertson, D. P. W. Jayatunga, B. G. D. N. K. De Silva, Vijay Jayasena

**Affiliations:** ^1^ School of Medical and Health Sciences, Edith Cowan University, Joondalup, Australia, ecu.edu.au; ^2^ Riddet Institute, Massey University, Palmerston North, New Zealand, massey.ac.nz; ^3^ Department of Biochemistry, Memorial University of Newfoundland, St. John’s, Canada, mun.ca; ^4^ Curtin Medical School, Faculty of Health Sciences, Curtin University, Perth, Australia, curtin.edu.au; ^5^ School of Biomedical Sciences, University of Western Australia, Crawley, Australia, uwa.edu.au; ^6^ Genetics and Molecular Biology Unit, Faculty of Applied Sciences, University of Sri Jayewardenepura, Sri Jayawardenepura Kotte, Sri Lanka, sjp.ac.lk; ^7^ Department of Nutrition and Food Science, Western Sydney University, Sydney, New South Wales, Australia, westernsydney.edu.au

**Keywords:** food products, health benefits, nutrients, processing, sorghum

## Abstract

Sorghum (*Sorghum bicolor* L.) is a member of the *Poaceae* family and is the fifth most important crop globally. Sorghum grains (SGs) are rich in health‐promoting macro‐ and micronutrients and phytochemicals. SGs are commonly consumed as food or as ingredients in food, especially in African countries. Therefore, food products such as ogi, bread and flour have been and are still being developed from SGs to provide nutritional and health benefits. However, the nutritional and prebiotic potential of SGs, especially the pigment pericarps, has not been fully exploited. This review describes micronutrients in different varieties of sorghum and the health benefits of sorghum consumption, especially its interaction with the human gut microbiota. It further provides a comprehensive update on the properties and health benefits of improved sorghum‐based food products. Finally, the influence of processing methods on SGs is summarised.

## 1. Introduction to Sorghum

*Sorghum bicolor L*., commonly known as sorghum, is a grain crop that belongs to the Gramineae family, which also includes cereals like wheat, maize and barley [1]. Originating from Africa, sorghum is highly valued for its resilience to harsh, dry climates, making it an important and sustainable food source, particularly in the context of climate change [1]. Since its domestication in Africa, sorghum has served as a staple food, traditionally used to prepare bread, rice and various beverages [2]. Nutritionally, sorghum is a rich source of essential macronutrients, including carbohydrates, protein and lipids. It also provides important micronutrients such as calcium (Ca), potassium (K), magnesium (Mg), iron (Fe), phosphorus (P), copper (Cu) and vitamin B6, contributing to its status as a valuable dietary component [2, 3].

Sorghum varieties can be distinguished by the pigment of their pericarp, which is the outermost layer of the sorghum kernel [3, 4]. This pigmentation is determined by the genotypic expression of *R* and *Y* genes, while the intensity of the pigment is modulated by the intensifier gene *I* [4]. This phenotypic diversity, particularly in grain colour variations of sorghum, is strongly linked to the physical characteristics and functional properties of the grains, which influence its end‐use in food and industrial applications.

The physical traits of sorghum, such as kernel hardness, size and pericarp thickness, vary notably among colour types. White and yellow sorghum grains (SGs) generally possess thinner pericarps and softer endosperms compared to red, brown or black varieties [5]. Darker‐coloured sorghum, especially red and brown types, often exhibits harder textures and higher bulk densities due to greater phenolic cross‐linking in the pericarp and aleurone layers [6]. The colour of SG is closely linked to pericarp structure, kernel weight and endosperm texture [7]. Brown hybrids (e.g., BRS 305 and 1167048) have a pigmented testa and softer endosperm, traits that are beneficial for bread‐making but also indicate higher levels of condensed tannins and phenolic compounds. In contrast, the white hybrid (CMSXS 180) lacks a pigmented testa and has a significantly harder endosperm, which makes it more resistant to milling and less digestible.

Further, insights from [8] showed that local Indonesian black and brown sorghum accessions had comparatively higher bulk density and tannin content, with physical features such as compact grain and thicker husk layers contributing to their antioxidant richness and resistance to spoilage. Meanwhile, red SGs, when processed via stone and roller milling, produced whole‐grain and fractionated flours with varied particle sizes and composition [9]. The roller milling system revealed that fractions derived from the outer bran‐rich layers (fine bran and dark endosperm fractions) had higher protein, fibre and polyphenol content, whereas the core endosperm fractions were richer in starch and exhibited finer particle sizes and lower water solubility indices. Together, these findings underscore the influence of SG pigmentation and physical architecture on processing outcomes and functional food potential. Dark‐coloured varieties generally offer superior bioactive content and antioxidant properties but present greater challenges in textural applications due to phenolic–protein interactions. Conversely, lighter‐coloured sorghum types may be less nutritious but more suitable for applications requiring smoother textures and bland flavour profiles.

Sorghum varieties, depending on the intensity of the pericarp pigment, have varying levels of bioactive compounds such as tannins, phenolic acids and flavonoids, which are known to promote anti‐inflammatory, antioxidant, anti‐diabetic, anti‐cancer and anti‐proliferative activities [2–4]. Phenolic acids are far more abundant in sorghum compared to other cereals, contributing to a superior antioxidant effect [3, 10]. Despite its reported high nutritional and health beneficial properties, the digestibility of sorghum is minimal. High tannin contents in some sorghum varieties can lower the digestibility of minerals because they form complexes with protein and Fe [3]. Nonetheless, some techniques can enhance the digestibility of sorghum, promoting its use for medicinal purposes. For example, ethnobotanical studies on sorghum often used a decoction of the grain from heating or boiling to alleviate symptoms of cancer, epilepsy, diarrhoea and stomach pain [11].

This review explores the nutritional constituents of SGs, with a particular focus on micronutrients and vitamins. Additionally, it provides an update on the biological properties of improved processed sorghum‐based food products. The effects of processing methods on health‐promoting nutritional constituents of sorghum are also described.

## 2. Nutritional Composition of *Sorghum bicolor*

*Sorghum bicolor* is known for its high concentrations of macronutrients, essential vitamins, minerals and phytochemical, contributing to its role as both a staple and functional grain. Genotypic differences, particularly in red and black varieties, are associated with higher protein, lipid and dietary fibre content, supporting glycemic regulation, lipid metabolism and satiety [8]. The carbohydrate fraction, primarily starch (75%–80%), contains high levels of amylose and resistant starch, which contribute to a low glycemic index and sustained energy release beneficial for metabolic health and diabetes management [8, 12]. Resistant starch and insoluble fibres in sorghum stimulate short‐chain fatty acid (SCFA) production through colonic fermentation, enhancing gut barrier function and reducing inflammation. Higher fibre content in pigmented varieties has also been linked to improved gastrointestinal function and reduced risk of colorectal conditions [13]. Though sorghum protein is limited in lysine, it is rich in branched‐chain amino acids (leucine, isoleucine and valine), and its kafirin proteins exhibit slow digestibility, promoting prolonged amino acid release and satiety [14].

Sorghum also stands out for its variety of secondary metabolites. There are high levels of phenolic compounds, particularly in pigmented genotypes. Brown and black varieties are rich in condensed tannins, 3‐deoxyanthocyanidins and phenolic acids (e.g., ferulic and p‐coumaric), which exert antioxidant, anti‐inflammatory and chemopreventive effects [7]. While tannins were once viewed as anti‐nutritional, current evidence highlights their functional roles in modulating oxidative stress and metabolic disorders, underscoring sorghum potential in health‐targeted food applications [15]. Readers are directed to our recently published work [16] for a detailed discussion on macronutrients and phytochemical contents of SGs.

### 2.1. Micronutrients

Micronutrients are health‐promoting molecules present in small amounts in foods, which are generally considered to be minerals and vitamins. Micronutrients are critical for synthesising hormones, enzymes and other substances required for normal growth, development and overall well‐being. A deficiency in micronutrients can lead to reduced energy levels, impaired cognitive function and compromised health [17].

SGs are rich in minerals that can be classified as macroelements (e.g., Ca, Mg, K and P) and microelements (e.g., manganese, zinc, Cu and Fe) (Table 1), making them an excellent nutritional source [18–24]. The microelement content of red sorghum (mg/100 g DW) is as follows: Ca (474.91), K (2.25), Mg (130.9), P (0.30), sodium (4.81), zinc (0.63), Fe (0.32), manganese (1.10) and Cu (1.25) [19]. However, earlier findings showed higher values for another red sorghum genotype: Ca (10.81), K (278.68), Mg (145.69), P (301.77) and sodium (4.21) [23]. These discrepancies may stem from differences in analytical methods, soil composition or environmental conditions. Comparisons between red and brown sorghum genotypes revealed variations in micronutrient content (mg/kg): Ca (4.75–5.55), K (4248.04–4663.45), Mg (1423.54–1445.86), P (3486.85), sodium (39.7–47.72), zinc (24.48–27.64), Fe (41.30–46.46), manganese (13.05–14.31) and Cu (4.99) [18]. Notably, brown sorghum exhibited a superior overall nutritional profile compared to the red genotype. Further analysis of 16 sorghum lines, including red, yellow and brown pericarp, showed that the estimated dietary intake of P, Mg, Cu, zinc and selenium from 100 g of these grains meets the recommended daily values for adult men and women [22]. These findings highlight the potential of these grains as key ingredients in the development of functional foods.

**Table 1:** Mineral content in sorghum grains.

	**Genotype/pericarp colour**						
	**Red, brown (mg/kg)**	**Red (mg/100** g**)**	**19 accessions (mg/100 g)**	**21 landraces; red, brown and yellow (mg/100 g)**	**16 varieties; red, brown and yellow (mg/100 g)**	**Red (mg/100 g)**	**20 improved varieties (mg/kg)**
Macroelements							
Calcium	4.75–5.55	474.91	74.79–110.99		80.11–189.93	10.81	50.713–511.69
Potassium	4248.04–4663.45	2.25	110.55–331.06		963.3–2322.58	278.68	577.19–3041.52
Magnesium	1423.54–1445.86	130.9	80.44–135.67		575.81–927.35	145.69	29.35–4399.49
Phosphorous	3486.85–3486.85	0.30	157.75–278.15		3080.87–6781.94	301.77	1342.96–3500.34
Sodium	39.7–47.72	4.81			2.96–5.39	4.21	25.97–185.25
Nitrogen			1.93–2.76				
Sulphur		0.25			725.31–1031.82		
Microelements							
Zinc	24.48–27.64	0.63		16.9–43			1.96–18.61
Iron	41.30–46.46	0.32		32–101			67.14–221.26
Manganese	13.05–14.31	1.10		9.21–20.2			4.72–11.39
Copper	4.99–4.99	1.25		1.5–5.3			0.13–3.46
Chromium		0.05		0–1.5			
	[18]	[19]	[20]	[21]	[22]	[23]	[24]

Additionally, traces of vitamins (mg/100 g) have been identified in red sorghum genotypes, such as thiamine (0.79), pyridoxine (0.10) and riboflavin (0.05) [25]. In contrast, very low concentrations (*μ*g/kg) were reported in other red pericarp genotypes, including thiamine (0.4), pyridoxine (0.53) and niacin (0.22) [26] (Table 2).

**Table 2:** Vitamins reported in red sorghum grains.

**Vitamins**	**Red (mg/100** g**)**	**Red (*μ*g/kg)**
Vitamin B1	0.79	0.40
Vitamin B6	0.10	0.53
Vitamin B2	0.05	nd.
Niacin		0.22
	[25]	[26]

Abbreviation: nd., not determined.

These findings further emphasise the nutritional diversity of sorghum genotypes and underscore its value as a sustainable source of micronutrients for addressing dietary deficiencies and supporting the development of functional foods.

## 3. Interaction With Gut Microbiota

The human microbiota is a fragile and dynamic ecosystem that comprises trillions of host‐specific fungi, viruses and bacteria [27]. The gut microbiota is dominated by four bacterial species, including Bacteroides, Firmicutes, Actinomycetes and Protobacteria, which are key elements that regulate human health [28]. Major factors affecting gut health include gut microbiome, gut barrier function, nutrient digestion and absorption, mucus layer, fecal pH, mucosal immune response and SCFA production [28]. A healthy microbiota may be disrupted by drugs, antibiotics, unhealthy foods and infection, leading to dysbiosis. Gut dysbiosis manifests in the form of a reduction in bacteria that synthesise SCFAs, lowered mucus production and increased intestinal permeability [29]. Prebiotic fibres and polyphenol contents of food crops remarkably influence the composition and function of the human gut microbiome and human well‐being [30]. Prebiotics include resistant starch, digestion‐resistant oligosaccharides and dietary fibres from plant sources [29]. There are increasing efforts to develop novel foods and novel food ingredients that can potentially modify the gut microflora to minimise susceptibility to diseases [31].

Human and animal studies have shown that SGs could act as prebiotics to enrich or modulate gut microbiota (Table 3). A 6‐week comprehensive study by de Sousa and colleagues in which dietary fibre was replaced with 50%–100% extruded brown pericarp sorghum flour (ESF) showed that the formulation modulated intestinal microflora, reduced inflammation and oxidative stress in obese rats [32]. The ESF reduced gut dysbiosis due to increased Bacteroides and decreased Firmicutes (18%–20%). Expression of inflammatory markers, Nuclear factor kappa‐light‐chain‐enhancer of activated B cells (0.08–0.12 times) and resistin protein (31.5%–36.8%) significantly declined while superoxide dismutase increased by 5.6–13.6 times. An innovative research developed a model for genetic analysis of 294 lines of IS3620 (from guinea sorghum) and BTx623 (a blend of Kefir and Caudatum) sorghum that influence fermentation patterns by the human gut microbiome [30]. The authors used grains from each sorghum line for automated *in vitro* microbiome screening (AiMS) to quantify the effects of the predigested grain on human gut microbes. First, the grain was milled and hydrolysed by acid and digestive enzymes before dialysis. The digest was incubated in a human stool microbiome from 12 donors, and abundances of microbial taxa were evaluated by 16S rDNA sequencing. In their results, *β*‐diversity indicated that 10 of the 12 microbiomes recorded significant differences in overall microbiome composition (*p* < 0.05). The different composition of the IS3620C and BTx623 parental lines caused different fermentation patterns of the human gut microflora. In addition, nine microbial clades demonstrated consistent, distinct responses to the parent sorghum lines in the microbiome of several donors [30]. Fermentation of IS3620C produced more abundant *Bifidobacterium, Coprococcus 1* and *Coprococcus 3* in the microbiomes of Donors 5, 4 and 7, respectively, whereas fermentation of BTx623 resulted in more abundant *Bacteroides*, *Parasutterela* and *Escherichia* in donors 7, 5 and 4, respectively [30]. This comprehensive study could be directly applied to other food crops to identify seed traits that influence the composition of the human gut microflora using a large population of stool donors.

**Table 3:** Interaction of sorghum‐based foods with gut microbiota.

**Genotype/country**	**Treatment/method**	**Findings/health benefit**	**Metabolites (*μ*mol/g cecum)**	**Reference**
Brown pericarp SC 319 (Brazil)	Extruded flour fed to eight obese rats for 8 weeks	↑ *Bacteroides* phylum ↓ *Firmicutes* phylum ↓ p65 NF‐*κ*B in liver, seric resistin, lipid peroxidation ↑ total antioxidant capacity of plasma, heat shock protein 72 ↓ intestinal dysbiosis, plasma SOD, CAT, GST and inflammation	Acetate, butyrate and propionate	[32]

Whole and refined sorghum flour (Japan)	Whole sorghum (WS), high amylose corn starch, *α*‐corn starch, refined sorghum were fed to six 7‐week‐old 130–160 g rats for 1 month	WS caused ↑ body weight, feed intake and serum lipid	Acetate, propionate and butyrate	[33]

Whole‐grain brown pericarp SC 319 (Brazil)	40 g extruded whole sorghum fed to 11 men for 8 weeks in a randomised control single‐blind trial	↓ weight, body fat ↓ genus levels of *Odoribacter*, *Dorea* and *Clostridium*‐*sensu_stricto 1*, Proctobacteria phyla ↑ CAG‐873 and *Turicibacter*	Acetic acid, propionic acid and butyric acid	[34]

IS3602 and BTx623 (USA)	Sorghum flour was milled, digested *in vitro* prior to colonic fermentation to screen and quantify effects on human gut microbiome using 16sRNA	Identified molecular components in sorghum that affect human gut microflora	Propionate, butyrate and valerate	[30]

Whole grain (China)	Extruded whole sorghum grain was digested *in vitro* The digest was incubated with human fecal sample for 24–48 h	↑ glycemic index (GI), SCFAs ↑ microbial diversity ↑*Megamonas, Prevotella, Bifidobacterium, Megasphaera, Parabacteroides* and *Phascolarctobacterium* ↓*Bilophyla*	Acetic acid, propionic acid and butyric acid	[35]

BRS 305 sorghum grain (Brazil)	A 10‐week single‐blind randomised controlled pilot study with 30 volunteers to evaluate effects of sorghum grain beverage on lipid profile, body weight and intestinal health	↑ probiotics ↓ Castelli index I	Valeric acid, acetic acid, propionic acid, butyric acid, isobutyric acid and hexanoic acid	[36]

Red sorghum (Iran)	Examination of effects of fermented red sorghum flour fed to AD mice	↓ oxidative stress, inflammation and acetylcholine esterase ↑ probiotics		[37]

*Note:* ↑increase, ↓decrease.

Note: ↑ increase and ↓ decrease.

In addition, another 8 weeks randomised controlled, single‐blind clinical trial in which 11 adult men were fed 40 g extruded SC319 whole brown sorghum showed no difference in SCFAs, *α* and *β* diversity compared to a whole wheat diet [34]. However, the SC319 diet decreased levels of *Odoribacter*, *Dorea* and *Clostridium-sensu_stricto 1* and the *Proctobacteria* phyla. *Dorea* is associated with glucose concentrations and prediabetes [38]. *Odoribacter* positively correlated with the expression of inflammatory cytokines such as IFN and TNF. The SC319 also enhanced weight loss, which the authors attributed to the 3‐deoxyanthocyanins and proanthocyanin contents [34]. However, the study involved a small number of participants and a short period of diet intervention.

Furthermore, the fibre content from sorghum influenced digestion and metabolic products [35]. The researchers employed a novel extrusion–expansion treatment on whole sorghum and other grains followed by simulated gastrointestinal digestion and colonic fermentation using human stool. The study indicated an increase in the total dietary fibre and a significantly higher population of probiotics, including *Megamonas*, *Prevotella*, *Bifidobacterium*, *Megasphaera*, *Parabacteroides* and *Phascolarctobacterium*. The identified SCFAs were acetic acid, propionic acid and butyric acid (Table 3). Recently, a new pilot human study in which 30 volunteers consumed extruded sorghum beverages for 11 weeks indicated a decrease in participants′ Castelli index I, waist volume and total visceral fat [36]. The authors associated the decrease to high contents of dietary fibre and tannins in the BR305 sorghum. Proanthocyanidins in sorghum regulate SREBP2, which modulates hepatic cholesterol homeostasis through ABCA1 and ATP‐Binding Cassette Transporter G1, thereby increasing HDL‐c levels [39]. In addition, *L. paracasei*‐supplemented sorghum showed decreased diarrhoea and enhanced stool consistency due to higher valeric acid production. Valeric acid inhibits nonalcoholic fatty liver disease in human cells [40]. A study on the therapeutic effects of nonfermented and fermented red sorghum on Alzheimer’s disease (AD) pathology in mice showed that fermented sorghum significantly improved all markers of AD [37]. However, the outcome cannot be extrapolated to humans.

Sorghum grains have emerged as promising dietary component for modulating gut microbiome composition due to its rich content of dietary fibre, resistant starches and polyphenols. These bioactive compounds serve as substrates for beneficial gut bacteria, promoting microbial diversity and metabolic health [41]. The indigestible components in sorghum, such as resistant starch and nonstarch polysaccharides, act as prebiotics, selectively stimulating the growth of health‐associated bacteria, including *Bifidobacterium* and *Lactobacillus* [41]. Additionally, sorghum′s polyphenols, particularly in pigmented varieties, exhibit antioxidant and anti‐inflammatory properties that further modulate gut microbiota by suppressing pathogenic bacteria while enhancing beneficial populations [42].

One key mechanism involves sorghum resistant starch, particularly amylose in nonwaxy varieties, which serves as a fermentable substrate for amylolytic bacteria such as *Roseburia* and *Faecalibacterium* [43]. The fermentation of amylose by these microbes increases butyrate production, a SCFA linked to anti‐inflammatory effects and improved gut barrier function [43]. In contrast, waxy sorghum, which lacks amylose, fails to support these beneficial microbes, leading to reduced microbial diversity and lower butyrate levels [43]. Another critical factor is sorghum condensed tannins, which are influenced by genetic variations in the *Tan1* and *Tan2* genes [30]. These tannins selectively promote the growth of beneficial bacteria, including *Faecalibacterium prausnitzii*, *Christensenellaceae* and *Roseburia*, while enhancing microbial diversity and SCFA production [30, 44]. The prebiotic effect of tannins is particularly notable in individuals with dysbiosis, as sorghum polyphenols may help restore microbial balance by stimulating butyrogenic taxa [42].

Sorghum genetic traits also play a role in microbiome modulation. Variations in starch composition, influenced by cell wall invertase genes, enhance amylolytic microbes like *Ruminococcaceae*, while high‐tannin genotypes promote *Faecalibacterium*, a bacterium associated with anti‐inflammatory effects [45]. Furthermore, extruded sorghum has been shown to increase beneficial genera such as *Turicibacter* and CAG‐873 while reducing potentially harmful bacteria like *Clostridium sensu stricto 1* and *Dorea* [34]. These shifts are attributed to sorghum slow digestibility and high antioxidant capacity, which enhance microbial fermentation [34].

## 4. Improved Sorghum‐Based Products

The utilisation of sorghum as an ingredient in food formulations has attracted considerable interest from researchers and the food industry. Sorghum‐based products have been extensively studied for their nutritional and potential health benefits. Currently, some novel methods are being employed to improve the nutritional profile and consumer acceptability of existing sorghum‐based food products as described below.

### 4.1. Extruded Snacks

Growing demand for health beneficial and nutritious snacks has triggered research on the production of extruded snacks from different blends of sorghum flour [46]. Although extruded snacks are rich in nutrients, the blends of the flour may impact the overall quality [47, 48]. Jenfa and co‐researchers formulated an extrudate from eight different blends of sorghum flour and orange‐fleshed sweet potato flour (Table 4). The blends with the highest content (80%) of orange‐fleshed sweet potato flour recorded the highest nutritional profile with increased vitamins A, B1 and C by 441.7%, 26.7% and 766.7%, respectively. Consumers preferred the blend′s gumminess, springiness and chewiness, with an overall acceptability of 7.15 [58]. In an attempt to produce a nutritionally balanced sorghum‐based cereal, [62] formulated an extruded product comprising date palm, yellow maize and sorghum flours in different proportions (10:90:0, 20:80:0, 30:70:0, 10:0:90, 20:0:80, 30:0:70 and 10:45:45). The date flour increased mineral bioaccessibility in the product.

**Table 4:** Improved sorghum‐based foods.

**Sorghum-based products**	**Formulation and outcome**	**Country**	**References**
Powder (flour)	• Gluten‐free flour with peak viscosity was 742.8 BU and average breakdown 419.9 BU • Suitable for sauces and baking • Rich in phenolic compounds with high antioxidant activity	Italy	[49]
Powder (flour) White/red sorghum	• Whole sorghum flour fermented with LAB plus skim milk • Whole flour with malted sorghum with skim milk present highest antioxidant activity (4.7 mM Trolox), antidiabetic activity (dipeptidyl‐peptidase IV inhibition 31.3%) and antihypertensive activity (ACE‐I inhibition 57.3%) • Whole flour with malted sorghum recorded highest antidiabetic activity (*α*‐amylase inhibition 38.1%) and antioxidant activity (1.6 mg AAE/mL)	Brazil	[50]
Powder (flour) White sorghum	• Malting by ultrasonication preserve nutritional value • Malting increased total phenolic and total flavonoid content (3.23 mg GAE/g and 3.05 mg QE/g) • Ultrasonication result in higher fibre and protein (4.53% and 11.74%)	South Africa	[51]
Powder (Bread) Red sorghum	• Sorghum fermentation with 20%–30% *L.acidophilus* • Better quality attributes: softer texture • Higher total phenolic content (97.75 mg GAE/100 g) and antioxidant activity (341.49 *μ*mol TEAC/100 g)	Turkey	[52]
Powder (bread) Kisra	• Kisra is fermented bread from sorghum flour • LAB increased TPC by up to 20% • *Pediococcus pentosaceus* fermented Kisra • Higher Kisra quality, safety and health benefits • Higher DPPH scavenging activity (84%), but lower TPC (1880 *μ*g/g)	South Korea	[53]
Powder (bread) White sorghum	• Blend of sorghum flour, corn starch and sourdough • Higher ash, crude protein and dietary fibre • Increase bread volume, aroma, taste, texture and overall acceptability (6.56)	Nigeria	[54]
Powder (bread)	• Gluten‐free bread was produced from tannin‐rich sumac sorghum bran. • Dietary fibre increased to 13.4% • Oxygen radical absorbance capacity was 61.6 *μ*mol TE/g	USA	[55]
Porridge (Ogi baba)	• Ogi baba is a creamy porridge from wet‐milled fermented sorghum • Sorghum ogi was fortified with soy flour • Crude protein, crude fibre, fat, ash and carbohydrates significantly increased • Magnesium, calcium and iron levels increased.	Nigeria	[56]
Fermented alcoholic beverage (*mahewu*)	• Blend of fermented sorghum and Bambara groundnut • Improved metabolite profile alkanes, esters and derivatives • Improved health benefits	South Africa	[57]
Extruded products (snack)	• Snack from sorghum and orange peel sweet potato • Vitamins A, B1 and C increased by 441.7%, 26.7% and 766.7%, respectively	Nigeria	[58]
Breakfast smoothie	• Breakfast smoothie was formulated from sorghum flour, carrot, mango, milk and honey • It is rich in dietary fibre (1.68%), calcium (560.46 ppm), iron (28.12 ppm) and vitamin A (679.3 IU/L) • Low in calorie (89.09 kcal/100 g) and fat (0.65%)	India	[59]
Cookies	• Enhanced aroma of 3D‐printed cookies • Improved dough elasticity (G ^′^)	USA	[60]
Biscuits (red sorghum)	• Sorghum‐based biscuit was fortified with Longhorn *Ruspolia differens* powder (RPD) • Ash, fat, protein and crude fiber increased by 133%, 37%, 118% and 573%, respectively • Lysine and digestible protein increased significantly	Uganda	[61]
Breakfast cereal (plus maize and date fruit)	• Improved functional properties: bulk density, swelling power, oil absorption capacity and water absorption capacity • Enhanced levels and bioaccessibility of minerals: potassium, sodium, calcium, iron and zinc	Nigeria	[62]

The mineral contents were K (253–351.65 mg/100 g), Ca (321.5–421.4 mg/100 g), sodium (10.88–18.67 mg/100 g), zinc (4.11–5.36 mg/100 g) and Fe (9.22–15.2 mg/100 g).

### 4.2. Biscuits/Cookies

Biscuits or cookies are ready‐to‐eat snacks made from flour, sugar and fat. Technologies are emerging to improve their properties to meet nutritional requirements to combat diet‐driven diseases. To this end, researchers have been partially substituting whole‐grain sorghum flour with wheat and other ingredients (Table 4).

Ronoh and colleagues formulated an enriched biscuit from sorghum flour by replacing sorghum–wheat flour with 5%, 15%, 20% and 40% *Ruspolia differens* powder (RPD). Protein digestibility corrected amino acid score (PDCAAS) of the formulated biscuit increased by up to 41%, with higher preference by consumers except for the 40% RPD‐fortified [61]. The improvement in the PDCAAS was associated with increased lysine content and protein digestibility. The dislike of the 40% RPD biscuit may be due to the unfamiliarity of consumers with the darker colour of the biscuits as the level of RPD increased. It is also likely that consumers disliked the RPD volatile aroma resulting from lipid oxidation, including carboxylic acids, alcohols and aldehydes. In another study, the authors formulated highly acceptable 10 biscuit varieties by substituting wheat and wheat–sorghum flours with 5%, 15%, 25% and 40% RPD [63]. Consumers’ acceptability of 5% RPD was the same as that of 0% RPD wheat–sorghum biscuit, while the preference level for all other biscuits, including 100% wheat, was the same. The least and most preferred fortified biscuits were 40% RPD and 5% RPD, with average likeness of 57% and 65%, respectively. Wheat–sorghum biscuits were crispier and crunchier, and the 5% RPD biscuit had a low fishy flavour [63].

Moreover, optimum conditions for producing highly nutritious and health‐promoting sorghum‐based biscuits were established by [64]. The authors blended grass pea, sorghum and orange peel flour (25:65:10, 15:80:5 and 35:50:15) at a temperature of 180°C–220°C for 15–25 min. The peel improved the antioxidant and nutritional fibre content of the biscuit with reduced antinutritional factors [64]. Orange peels have high dietary fibre content and antioxidant activities [65] and baking has been shown to diminish levels of phytic acids in foods [66].

### 4.3. Bread

The increasing demand for gluten‐free (GF) products stems from medical reasons and consumer concerns about the health implications of food products. Producing GF bread with high nutritional and desirable sensory qualities is very challenging [55]. Strategies for producing GF bread with enhanced properties include technological modifications and ingredient optimisation [67]. By optimising the proportion of tannin‐containing sumac sorghum bran, water and gum, Ardoin and co‐researchers produced GF bread from 14.2% sorghum bran, 145% water and 1% gum. Due to the high amount of fibre and antioxidant compounds (flavonoids and phenolic acids) in the sorghum bran [4, 68], the tannin‐rich sumac sorghum increased the fibre content and *in vitro* antioxidant capacity of the final product to 13.4% and oxygen radical absorbance capacity of 61.6 *μ*mol TE/g, respectively [55]. The sensory properties of the bread were not affected.

The choice of starter cultures used for flour sourdough fermentation also influences the quality of the GF bread. A recent study on the effects of sourdough fermentation and nutritional enrichment on the quality of GF sorghum used *Saccharomyces cerevisiae*, *Lactobacillus acidophilus* and *Lactobacillus casei* as starter cultures [52]. The authors reported that SG breads made with 20% and 30% *L. acidophilus* had a desired softer texture, higher total phenolic content (97.75 mg/100 g) and antioxidant capacity of 341.49 *μ*mol TEAC/100 g. During fermentation, *Lactobacillus* strains produce several antioxidant bioactive compounds through specific complex anabolic pathways [52, 69, 70]. Olojede and colleagues also prepared sourdough from 20% of *Weissella confusa* SD8, *Pediococcus pentosaceus* LD7 or *P. pentosaceus* SA8. *P. pentosaceus* SA8 yielded a product with the highest dietary fibre, total phenolic content (TPC) and DPPH radical scavenging activities [54]. Other strains, including *P. pentosaceus* K‐B21 and *P. pentosaceus* K‐B01, increased the DPPH radical scavenging activities in Kisra bread to 84.16% with 1851.20–1880.60 *μ*g/g TPC [53]. Meanwhile, *Weisella cibaria*, *Levilactobacillus brevis*, *Limosilactobacillus fermentum* and *Lactiplantibacillus plantarum* significantly increased the TPC in Kisra by up to 20% [53]. The increased TPC may be attributed to improved bioavailability of the polyphenols caused by the hydrolytic activities of cell wall–degrading fermentation microorganisms [71, 72]. Fermentation results in the accumulation of antioxidant free phenolic compounds and metabolites, leading to increased DPPH radical scavenging activities [53].

### 4.4. Nonalcoholic Beverages

Several nonalcoholic beverages have been produced from sorghum, including *mahewu* and smoothies. *Mahewu* is a traditionally fermented nonalcoholic beverage native to South Africa, widely used as baby food for weaning and energy drinks for adults [73]. Preference of consumers for healthy foods offers sorghum, a nutrient and polyphenol‐densed grain, as an alternative ingredient for producing *mahewu* [74]. Recently, [57] produced *mahewu* from a blend of sorghum flour and 20/30% Bambara groundnut (BGN). Sorghum–BGN *mahewu* present improved metabolites including alcohols (17%), alkanes (12%), esters (21%), acids and derivatives (9%). Through the phosphoketolase pathway, lactic acid bacteria (LAB) break down amino acids or carbohydrates to produce alcohols [75]. LAB also synthesises various esters from the alcohols [76]. An earlier report showed that crude fibre (7.7%) and protein contents (7.9%) of sorghum–BGN *mahewu* blends were slightly higher than the control (6.1% and 5.7%), accompanied by an increase in levels of threonine, isoleucine, histidine, leucine, lysine and methionine [77]. The increase was due to the presence of protein and amino acid–rich (24%) BGN. A new study has introduced a convenient grab‐and‐go functional breakfast smoothie from carrot, mango, honey, milk and sorghum flour. The mixture was blended with sugar, pectin and sodium citrate before being adjusted to pH 4.2, followed by preheating at 65°C, homogenisation and pasteurisation at 95°C for 5 min. With an 88% acceptance level, the product had high dietary fibre (1.68%), Ca (560.46 ppm), Fe (28.12 ppm) and vitamin A (679.3 IU/L), but low in calories (89.09 kcal/100 g) and fat (0.65%) [59]. Without preservatives, a 200‐mL glass bottle stored at 4°C and 30°C had shelf lives of 75 and 60 days, respectively. The functional properties of the product could be attributed to the nutritional profile and antioxidant content of the key ingredients.

### 4.5. Sorghum Flour

A novel study by [51] employed conventional (fermentation and malting) and ultrasonication to improve the quality attributes of sorghum flour blended with *Moringa oleifera* and mopane. The ultrasonication of the flour blend resulted in higher TPC (3.23 mg GAE/g), TFC (3.05 QE/g), fibre (13.38%) and protein (4.53%). The flour and resulting products could help address malnutrition and health challenges in different age groups of consumers.

## 5. Influence of Processing Methods on Sorghum Composition

The influence of processing methods on sorghum composition is multifaceted, wherein the application of traditional and innovative techniques yields substantial improvements in the nutritional profile. The improvement is particularly evident in the bioavailability of essential nutrients, as well as in the modification of lipid composition. Some conventional methods like soaking, ash treatment, germination, fermentation, cooking, steaming and roasting have proven effective in altering the fatty acid profile and augmenting nutrient availability [78]. However, recent advancements, such as microwave roasting, nixtamalisation and irradiation, have further optimised these benefits, thereby rendering SGs even more nutritious and functional. Although these processing techniques improve the quality of sorghum‐based foods, making them a more valuable nutrient source; the effect of different processing methods on sorghum composition remains to be explained. This complexity underscores the necessity for continued investigation into the specific impacts of each processing method on the overall nutrient density and health benefits conferred by SGs [79, 80].

### 5.1. Soaking

Soaking is widely recognised as a low‐cost technique employed in the food industry to enhance the quality of raw materials, particularly in grains such as sorghum. This process facilitates the absorption of water, which activates enzymes involved in the digestion of grain nutritional constituents. The penetration of water reduces antinutritional factors present in the soaked grains [81]. Additionally, water penetration through the cell walls improves the accessibility of total flavonoids, a type of membrane‐bound phenolic compound, for absorption or chemical analysis [82]. Furthermore, soaking can improve the bioavailability of essential minerals such as Fe and zinc, thus contributing positively to the nutritional profile of sorghum [83]. However, while the soaking process can enhance certain nutritional aspects, it may also result in the leaching of soluble minerals and bioactive compounds, thereby diminishing the overall nutritional quality of SGs [84]. For instance, soaking SGs in distilled water for extended periods has been shown to significantly reduce levels of total phenols, flavonoids and antioxidant activity, likely due to the solubilisation of these compounds into the soaking water [76, 85]. The duration of soaking is critical; prolonged soaking can exacerbate the loss of beneficial nutrients, as evidenced by studies [86] that show longer soaking times increase the diffusion of nutrients out of the grains. To mitigate these negative effects, researchers have suggested combining soaking with other treatments, such as germination, which can enhance nutrient availability while minimising nutrient loss [87]. Overall, while soaking presents both advantages and disadvantages, its application can be optimised through careful management of soaking duration and conditions to maximise the nutritional benefits of grains [78]. However, this process necessitates a nuanced understanding of the interplay between soaking time and nutrient retention, because improper practices may inadvertently lead to detrimental outcomes.

### 5.2. Germination

Germination plays a pivotal role in enhancing the nutritional profile and palatability of SGs by activating various biochemical pathways that improve their nutritional value. During germination, intrinsic proteases become active, enhancing protein digestibility and increasing the overall nutritional quality of the grains. This process can lead to a significant increase in dietary fibre content, with some studies reporting notable improvements due to the metabolic activities involved in sprouting [84, 88]. Additionally, sensory properties are enhanced through improved protein solubility and digestibility [84]. Furthermore, antinutritional factors such as phytates, which are common in cereals, are broken down more effectively due to the activation of several digestive enzymes. This enhances the bioavailability of essential minerals like Fe and zinc [89]. Germination also modifies the functional properties of sorghum flour, leading to improved water and oil absorption capacities. This is accompanied by enhanced emulsifying and foaming properties. For instance, the water absorption capacity of germinated sorghum flour was significantly higher than that of nongerminated flour, indicating improved hydration properties that are advantageous for various food applications [90]. However, it is crucial to recognise that germination can induce the production of potentially deleterious compounds, such as hydrogen cyanide (HCN), in certain sorghum varieties. Research conducted by Yang et al. [91] reported that germination could substantially elevate HCN levels, thereby necessitating meticulous management of germination conditions to mitigate this risk. However, the extent of these improvements may vary, highlighting the importance of germination in optimising the nutritional potential of grains and it also introduces specific risks that must be addressed through appropriate processing techniques.

Integrating germination with other methods, such as alkaline soaking or nixtamalisation, has been proposed to maximise its nutritional advantages while minimising potential risks. Malting, an intricate process involving soaking and germinating of SGs, also plays a crucial role in enhancing their nutritional profile. This process reduces antinutritional factors while increasing the availability of essential amino acids and minerals [92]. Malting can also improve the sensory properties of sorghum, making it more palatable [93]. However, the nutritional composition of sorghum can vary depending on the malting duration and conditions, emphasising the need for optimised processing protocols to maximise health benefits [92].

### 5.3. Fermentation

Fermentation plays a critical role in enhancing the nutritional profile of sorghum‐based foods through the action of LAB and yeast. Fermentation improves protein content and digestibility by breaking down the protein–fibre matrix, thereby facilitating the access of digestive enzymes to the protein [94, 95]. This process also enhances the ratio of essential amino acids while increasing the digestibility and bioavailability of essential nutrients [96]. For instance, lactic acid fermentation has been shown to elevate protein content from 11.36% to 12.13% and enhance the essential amino acid ratio from 38.06% to 43.45% [94, 97]. This improvement arises from the synthesis of proteins by the fermenting microflora and the liberation of free amino acids [94, 98]. Furthermore, fermentation reduces fat and ash content, indicating microbial activity that degrades lipids and leaches water‐soluble minerals into the fermentation medium [94, 99]. Moreover, some findings highlight the augmentation of beneficial phenolic compounds, including *γ*‐aminobutyric acid (GABA), which is associated with various health benefits, such as antioxidant potential, reducing oxidative stress and serving as a functional ingredient in the management of diabetes mellitus [98, 100]. The process also dismantles antinutritional factors such as tannins and phytates, which are linked to the malabsorption of sorghum grain nutrients, thereby significantly boosting its nutritional value. Studies indicate that fermentation can reduce tannin content by 52.7% to 56.9% and phytic acid levels by over 50% [101, 102]. The enzymatic activities of phytase and tannase, released during fermentation, further contribute to the breakdown of these compounds, improving mineral bioavailability [103, 104]. Although fermentation serves as a valuable processing technique, it not only improves the nutritional quality of sorghum but also enhances its potential as a functional food.

### 5.4. Cooking

Cooking treatments (e.g., boiling, steaming and roasting) play a crucial role in enhancing the nutritional profile of SGs by reducing antinutritional factors and improving the bioavailability of essential nutrients. Research indicates that these cooking methods significantly decrease levels of antinutrients such as phytic acid and tannins, which are known to inhibit mineral absorption [101, 105]. For example, wet cooking treatments lasting 2 h have been shown to effectively reduce phytic acid and tannin contents, likely due to thermal breakdown and leaching into the cooking water [76]. Furthermore, cooking can enhance the availability of minerals like Fe and zinc by 20%–44% and 4%–29%, respectively, although this effect is variable. The increase is attributed to the degradation of phytate complexes that bind these minerals at high cooking temperatures [101, 106]. However, cooking SGs can reduce *in vitro* protein digestibility due to the cooling effect, which leads to formation of resistant starch. This starch interacts with proteins, making them less accessible to digestive enzymes [101]. Additionally, cooking induces endogenous factors, such as trypsin inhibitors and transformations in protein hydrophobicity, that affect protein structure and digestibility [101]. The method of preparing sorghum for cooking also influences its bioactive traits. For instance, steaming has been shown to increase the total phenolic and flavonoid content in SGs by disrupting cellular structures, thereby enhancing antioxidant activities [76, 107]. Roasting, on the other hand, improves the release of bound phenolic compounds and enhances antioxidant properties by breaking down cell walls and converting polymeric phenolics into more extractable forms [76, 106]. The impact of cooking on the bioactive compounds in sorghum is complex, depending on the specific cooking technique and the type of SG. While cooking can result in the loss of certain phenolic compounds, it can also lead to an increase in others, ultimately augmenting the antioxidant capacity of the cooked dish [107]. Thus, the choice of cooking method is critical for maximising the health benefits and nutritional value of sorghum [101, 106].

### 5.5. Decortication

Decortication, the process of removing the outer layers of SGs, significantly impacts the distribution of fatty acids and bioactive compounds, thereby influencing the nutritional quality of SGs. This method leads to a substantial reduction in antinutritional factors: the total condensed tannins and phytic acid [108], which can disrupt nutrient absorption. Phytic acid binds essential minerals like zinc and Fe, while tannins bind enzymes and proteins. Hence, decortication enhances the nutrient availability and digestibility [109], accompanied by the concentration of flavonoids and phenolic acids with strong antioxidant properties [85]. However, decortication results in loss of both proximate composition and essential nutrients and minerals. The effects of decortication on functional properties include the suitability of whole grain flour for emulsion‐type food formulations and an improvement in the colour properties of flour after decortication [108]. With advanced decortication techniques, the nutritional benefits and health‐promoting properties of sorghum can be maximised, highlighting its role in wholesome and nutritious diets.

### 5.6. Alkalinisation

Communities in southwestern Uganda and Burundi are using wood ash, an alkaline substance, as an effective and affordable solution to reduce the high tannin levels found in specific sorghum cultivars, such as red sorghum and bird‐resistant varieties. In this method, SGs are soaked in a slurry of wood ash and allowed to germinate for about 4 days. This process significantly lower tannin content while maintaining the nutritional quality of the grains [110, 111]. This treatment can reduce tannin levels by up to 50.2%, likely due to the hydrolysis or depolymerisation of tannins in the alkaline environment created by the wood ash, or through the interaction of tannins with cations such as K, sodium and Ca present in the ash [111, 112]. Moreover, wood ash treatment methods enhance the nutritional value of the treated SGs, achieving a 39.5% reduction in tannin content and a 7.3% increase in metabolisable energy [111]. This improvement is considered highly beneficial for increasing the nutritional uptake of essential nutrients while enhancing protein digestion due to the reduction in disruptive tannin concentrations [78, 111]. Additionally, feeding trials with rats and chicks have shown that germinated SGs treated with wood ash improve growth rates and feed efficiency. This further emphasises the practical benefits of this treatment for enhancing animal nutrition [78, 111]. Overall, the use of wood ash provides a cost‐effective approach to mitigating tannin‐related challenges in sorghum, while positively impacting its nutritional quality for both human and animal consumption.

### 5.7. Nixtamalisation

Nixtamalisation significantly enhances the nutritional value of SGs for human consumption through a thermo‐alkaline hydrolysis process that utilises lime (Ca(OH)_2_) or sodium hydroxide (NaOH). This method enriches the amino acid content and reduces the presence of detrimental elements, such as tannins and phytates, which hinder nutrient absorption [79, 105, 113]. Studies have shown that nixtamalisation effectively decreases condensed tannins by up to 90% in red sorghum while preserving beneficial phenolic compounds and antioxidant activity [107, 114]. The significant reduction in tannins primarily results from the removal of the pericarp during the nixtamalisation process, where these compounds are predominantly located [115, 116]. Additionally, the alkaline environment created during the process disrupts the molecular structure of phenolic compounds, causing them to leach into the cooking water [117, 118]. Beyond reducing antinutritional factors, nixtamalisation enhances the bioaccessibility of proteins, improving their digestibility during intestinal digestion, which is crucial for nutrient absorption [99]. The indigestible fraction of nixtamalised SGs has also been suggested to contribute to colon cancer prevention by influencing apoptosis and inflammation pathways [76]. Furthermore, nixtamalisation improves the functional properties of sorghum flour, such as water and oil absorption capacities, which are vital for food processing and product development [119, 120]. Overall, nixtamalisation not only enhances the nutritional quality of sorghum but also supports the development of healthier food products, positioning it as a valuable technique in the food industry [121, 122].

### 5.8. Irradiation

The application of gamma and electron beam irradiation has significantly enhanced the nutritional value of SGs by effectively reducing antinutritional factors. Compounds such as phytic acid and tannins, which are associated with the inhibition of mineral absorption and protein digestibility, are substantially diminished when exposed to gamma radiation [123, 124]. Studies have reported reductions in phytic acid levels ranging from approximately 39% to 90% and tannin contents from 28% to 86%, without adversely affecting the proximate composition or mineral bioavailability of SGs [78]. The reduction of these antinutritional factors promotes improved nutrient digestion, as evidenced by enhanced *in vivo* digestibility of dry matter and crude protein after irradiation treatments. Furthermore, the application of gamma irradiation, particularly at doses such as 10 kGy, has been shown to improve the properties of weaning porridge made from SGs while reducing *α*‐ and *β*‐amylase activities, which is important for starch digestion [125]. Similarly, electron beam irradiation, though its mechanisms are not as well understood, also contributes to improved nutritional profiles of SGs by enhancing antioxidant activity and increasing polyphenol content [126]. The combined effects of these two irradiation processes not only elevate the nutritional value of SGs but also improve food safety by decontaminating the grains. Together, these irradiation methods offer promising applications for both the nutritional enhancement and preservation of SGs, supporting better health outcomes by making these grains more digestible and nutritionally accessible.

## 6. Future Directions and Research Opportunities

Sorghum plays a pivotal role in sustainable agriculture and nutrition, addressing global food security and health challenges. As a GF cereal with rich fibre content, bioactive compounds and adaptability to diverse environments, sorghum is a valuable option for promoting health.

The integration of bioactive compounds into food formulations presents opportunities for producing functional foods that offer targeted health benefits. Developing a comprehensive database of sorghum phenolic compounds is crucial for both breeding programs and industrial applications. Advanced analytical techniques, such as mass spectrometry and nuclear magnetic resonance, can facilitate the discovery of novel bioactive compounds in sorghum. Future research should focus on the bioavailability, metabolic transformations and synergistic effects of these compounds in the human body to confirm their health‐promoting properties.

Investigating the antiviral properties of sorghum is particularly relevant, especially in light of pandemics such as COVID‐19. Nutraceuticals derived from SGs could serve as adjunct treatments for viral diseases and immune system enhancement, necessitating further *in vitro* and *in vivo* studies.

By integrating knowledge from genetics, food science and public health, researchers can maximise biological potential of sorghum grains. The future of sorghum research depends on leveraging genomics and advanced processing technologies to enhance its nutritional profile and increase consumer acceptance. Efforts supported by policy advocacy and public awareness campaigns could establish sorghum as a staple for healthier and more sustainable diets globally.

## Conflicts of Interest

The authors declare no conflicts of interest.

## Funding

No funding was received for this manuscript.

## Data Availability

Data sharing is not applicable to this article as no datasets were generated or analysed during the current study.
